# Biomolecules in Dental Applications: Randomized, Controlled Clinical Trial Evaluating the Influence of Hyaluronic Acid Adjunctive Therapy on Clinical Parameters of Moderate Periodontitis

**DOI:** 10.3390/biom11101491

**Published:** 2021-10-09

**Authors:** Iwona Olszewska-Czyz, Kristina Kralik, Jelena Prpic

**Affiliations:** 1 Department of Periodontology and Oral Pathology, Dental Institute, Medical Faculty, Jagielonian University, 31155 Krakow, Poland; 2 Department of Medical Statistics and Medical Informatics, Medical Faculty Osijek, University Josip Juraj Strossmayer of Osijek, 31000 Osijek, Croatia; kristina.kralik@gmail.com; 3 Department of Oral Medicine and Periodontology, Faculty of Dental Medicine, University of Rijeka, 51000 Rijeka, Croatia; jelena.horvat.prpic@gmail.com

**Keywords:** hyaluronic acid, non-surgical periodontal debridement, periodontitis

## Abstract

The biological activity of hyaluronic acid (HA) has been well-researched during the past decades; however, there are few randomized, controlled trials of its clinical effects in periodontal therapy. The purpose of this study was to evaluate the effect of hyaluronic acid on the principal parameters of periodontal healing. A specific, commercially available formulation designed and registered for professional dental application, composed of 16 mg/mL of cross-linked and 2 mg/mL of non-cross-linked HA, was used as an adjunctive to non-surgical periodontal therapy, and clinical parameters were evaluated after 3 months. The addition of HA to periodontal therapy demonstrated more favorable clinical results regarding reduction in inflammation, measured by bleeding on probing (−6% compared to the control group) and gain in periodontal attachment (1 mm more than control group), while it had no effect on probing depth reduction. No side effects were reported. Our study demonstrated that HA is a safe and easy-to-use biological agent; due to its wide array of properties, it may significantly improve the results of periodontal therapy. However, more long-term studies are needed to investigate whether these favorable effects remain over time.

## 1. Introduction

### 1.1. Background

Periodontitis is a chronic, inflammatory disease leading to pathological loss of tissues supporting the teeth. It has a multifactorial pathogenesis and involves complex interactions among dysbiotic plaque and destructive immune responses [1]. Epidemiological studies showed increased frequency and severity of periodontitis, which affects almost 50% of the population, with a tendency to expand with age [2]. It has also been linked to various systemic conditions such as cardiovascular disease, diabetes mellitus, rheumatoid arthritis and metabolic disease [3]. Some studies suggested that periodontitis plays a causal role in the initiation or aggravation of some of the above general disorders, most likely by stimulating an immune-inflammatory response [4]. If periodontitis is treated by professional bacterial biofilm control, it can be slowed down or stopped in most cases; however, if any factor affects either the local environment or the host response, progression of the disease and deterioration of the therapy response may occur [5]. On the other hand, some clinical studies have shown that periodontal treatment could improve other systemic conditions, for example, by better glycemic control in diabetic patients or by reducing serum inflammatory biomarkers such as C-reactive protein [3,4,5].

Periodontal diagnostics is currently based on clinical criteria, and the keystone of therapy is a non-surgical approach (professional plaque removal and subgingival instrumentation). Mechanical biofilm control leads to reduction in probing depths as well as clinical attachment gain. In some cases, supportive, adjunctive, local antimicrobial treatment is applied [6]. As the use of some locally administrated drugs is restricted to certain clinical situations due to their side effects, clinicians are still seeking additional therapy tools, such as dual-wavelength or photodynamic diode lasers, as well as new agents, which could be of benefit in periodontal therapy [7,8,9,10].

Hyaluronic acid (HA) is one of the local substances recently used as an addition to non-surgical periodontal treatment due to its biocompatibility, biodegradability, and properties of wound healing, rather than its antimicrobial impact [7,8,11]. HA is a biological molecule that can be found in many different tissues in the human body and is widely used in biomedicine. Studies have shown that hyaluronic acid can be found in gingivae, periodontal ligaments, cementum, alveolar bones, and in unstimulated saliva with a concentration of 148 to 1270 ng/mg protein [7,8]. It is an important component of the extracellular matrix and plays a significant role in cell migration and proliferation, which contributes to wound healing, tissue regeneration, and immunomodulation [11,12]. HA seems to be successful in the therapy of various medical problems; however, the dental application of this agent is relatively new [13,14,15]. As the results of some studies have suggested that HA may play bacteriostatic role [16], has the ability to interact with stem cells [17,18], and has tissue regeneration potential [7], it has been employed as a component of different products [13].

Hyaluronic acid concentration is tissue-dependent, and its properties are determined by molecular weight. In general, high-molecular-weight HA (HMW > million Da) has immunosuppressive and anti-angiogenic properties, medium-size HA (HMW form 2 × 10^4^ to 1 million Da) influences embryogenesis, wound healing, and regeneration, and small HA molecules (HMW from 6 × 10^3^ Da to 2 × 10^4^ Da) contribute to pro-inflammatory, angiogenic, and gene expression effects. The majority of HA-based agents used in periodontal therapy contain high molecular weight HA [8]. It was reported that high-molecular-weight HA products do not prolong inflammation, impair the healing process, or cause excessive metalloproteinase (MMP) expression at the repair site in gingival tissue [8]. Other studies revealed that HMW hyaluronic acid increases the proliferation of human periodontal ligament (PDL) cells and maintains their high viability [18]. Hyaluronic acid in dentistry has been recently used in the treatment of mucogingival defects and residual periodontal pockets; in improving wound healing, sinus lifting, bone grafting, and socket preservation; or as a physical barrier between soft and hard tissues in procedures such as regenerative and plastic surgery, and in local therapy of various types of lesions within the oral mucosa [8,19,20]. To the best of our knowledge, there are very few studies on HA adjunctive therapy of periodontitis, and none of them were conducted on a group of moderate periodontitis cases, for which this procedure would have been the first periodontal treatment attempt. Moreover, there are few hyaluronic-acid-based products registered and tested in randomized, clinical trials for applications in periodontal procedures. Furthermore, most of the studies failed to report the exact type and molecular weight of the agents used.

Considering all of the above aspects, a hypothesis was raised of the potential influence of hyaluronic-acid-based gel (with defined molecular properties), used as a local adjunctive to non-surgical periodontal therapy, on treatment outcomes of localized, moderate periodontitis.

### 1.2. Objectives

The aim of the study was to evaluate the impact of hyaluronic-acid-based gel as a local delivery agent in therapy of localized moderate periodontitis, by clinical parameters’ assessment. The main research objective was to investigate whether additional use of HA affects treatment outcomes, and to analyze eventual differences with the control placebo group.

### 1.3. Trial Design

**Figure 1:**
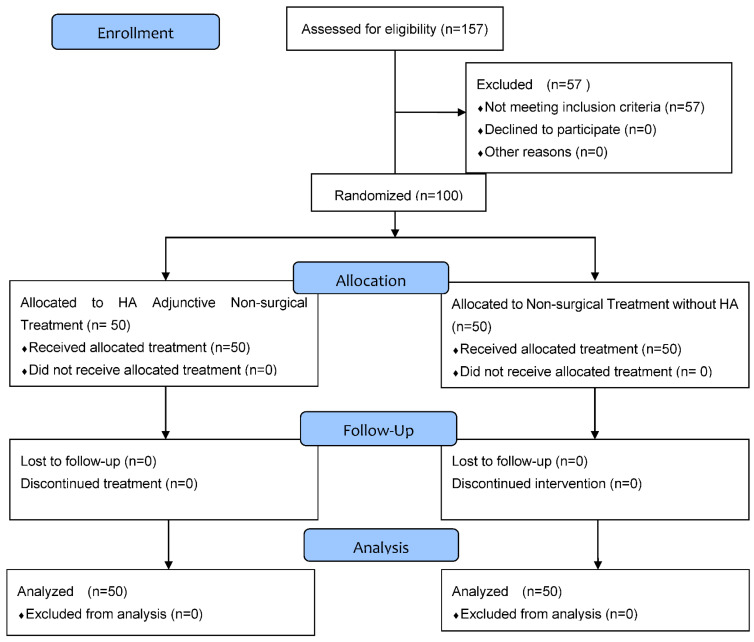
CONSORT 2010 flow diagram.

The trial was a 3 month, single center, prospective, randomized, controlled, single-blinded clinical trial conducted at the Periodontology Department of University Dental Clinic in Cracow, Poland. The study was performed in accordance with the Declaration of Helsinki. All the participants gave informed consent to participate in the study. Official approval from the Jagiellonian University Ethics Committee was obtained (No. 122.6120.132.2015). The participants were enrolled during the periodontal appointments and the trial design follows the CONSORT guidelines (Figure 1).

### 1.4. Randomization and Blinding

Patients enrolled in the study received code numbers. Randomizing software was used to randomly allocate patients to one of the two groups (non-surgical treatment only study group, and non-surgical treatment with adjunctive hyaluronic acid (HA) treatment control group; allocation ratio: 1:1) was used [21]. Participants were blinded to which treatment group they were assigned, as the hyaluronic acid was applied by an anesthetic syringe, whereas the non-surgical treatment alone also involved using an anesthetic syringe containing anesthetic agent.

## 2. Materials and Methods

### 2.1. Participants

One hundred, generally healthy, adult participants aged from 25 to 65 years (51% women) were enrolled in the study. The subjects were recruited from patients who had completed the first step of periodontal therapy (oral hygiene instructions and supragingival cleaning) no later than four weeks before enrollment in the study, and presented with an approximal plaque index (API) of less than 25%. To be included in the study, the patients had to demonstrate localized, moderate periodontitis with a minimum of two sites, and with a periodontal probing depth (PPD) equal or greater than 4 mm. Radiographs were used to confirm the diagnoses. A diagnosis of periodontitis was based on clinical and radiological examination, in accordance with the 2017 World Workshop on the Classification of Periodontal and Peri-Implant Disease and Conditions [22]. None of the participants had taken any antibiotics for the past 6 months, nor any non-steroidal anti-inflammatory drugs, corticosteroids, or multivitamin supplements within the 3 months prior to enrollment. They had to be nonsmokers (for a minimum of 5 years), and free from caries, epithelial dysplasia, and inflammatory lesions of the oral mucosa. Those with a history of rheumatic disorders, Sjögren disorder, enteritis, asthma, or sinusitis were also excluded from participation in the study. Pregnancy and having received periodontal treatment in the 6 months prior to the study were also added to the exclusion criteria.

### 2.2. Data Collection

Data were collected at baseline and after 12 weeks. Medical history, medication use, demographics, and oral hygiene routine were recorded. The periodontal clinical parameters were measured.

### 2.3. Clinical Parameters

A single periodontal examiner performed the following oral examination: approximal plaque index (API) [23], bleeding on probing (BoP) [24], periodontal probing depth (PPD), and clinical attachment level (CAL). The instrument used was a periodontal probe (PCP-UNC 15, Hu-Friedy, Chicago, IL, USA).

### 2.4. Intervention

The hyaluronic acid (HA) adjunctive treatment study group (*n* = 50) received non-surgical periodontal therapy, including subgingival instrumentation, followed by HA application to the existing pockets, while the control group (*n* = 50) received only non-surgical periodontal therapy without HA application [25]. The non-surgical instrumentation for both groups took place during one session at baseline and after six weeks, as the HA-based gel was applied to existing pockets in the study group for the second time after six weeks. All patients were followed-up after 12 weeks and, after the trial, patients were referred for either follow-up periodontal care or additional treatment, as needed. Consecutive, supportive periodontal therapy was provided.

### 2.5. Hyaluronic Acid Gel

The hyaluronic acid used in this clinical trial was a commercially available product, with defined composition and physical properties (Hyadent BG^®^, BioScience GmbH, Dummer, Germany). The gel was a mixture of 16 mg/mL cross-linked HA and 2 mg/mL non-cross-linked HA; the average molecular weight of the cross-linked HA as well as for the non-cross-linked HA was 1 million Dalton. The HA in this product was obtained by bacterial fermentation using *Streptococcus zooepidermicus*. The cross-linking process, using BDDE (1,4-butanediol diglycidyl ether), was performed in an alkaline pH, which resulted in formation of ether bonds. The degree of cross-linking was in the range of 0% to 20%.

### 2.6. Safety Monitoring

Oral symptoms were recorded at baseline, after six weeks and after twelve weeks. During the follow-up examination, the patients were asked if they had experienced any diverse events.

### 2.7. Statistical Methods

Categorical data are presented in absolute and relative frequencies. The differences between categorical variables were tested by the Fisher exact test. Normality of distribution for numerical variables was tested by using the Shapiro–Wilk test. Since the distribution was not normal, nonparametric tests were used, and numerical data are presented with both median and limits of interquartile range. Differences in numerical variables between independent groups were tested by the Mann–Whitney U test, with a 95% confidence interval (CI). Differences in values of continuous variables before and after applied therapy were tested by the Wilcoxon test. Correlation assessment was presented through the Spearman coefficient of correlation. Level of significance was set to alpha = 0.5. In order to obtain the effect size of 0.5 for the determination of the difference in numerical variables between the two measurements, with the level of significance set to 0.05 and the power set to 0.9, the calculated minimum required sample size was 44 subjects per group. Statistical analysis was performed with MedCalc^®^ Statistical Software version 19.6 (MedCalc Software Ltd., Ostend, Belgium (https://www.medcalc.org; accessed on 9 May 2020) and SPSS (IBM Corp. Released 2013. IBM SPSS, Ver. 21.0. Armonk, NY, USA).

## 3. Results

### 3.1. Adverse Events

No cases requiring rescue therapy were reported.

### 3.2. Results

The median age of study participants was 51 years in the control group and 52 years in the HA study group. There were slightly higher values of periodontal probing depths (PPD) in the HA study group before treatment, whereas no significant differences were observed for other parameters (medium PPD 4.2 mm in control group vs. 4.75 mm in the study group, *p* = 0.001). Table 1 presents differences between the clinical parameters of the groups at baseline and after therapy was completed. Statistically significant differences were observed between the two groups for BoP and CAL in favor of the HA study group, but no differences were found for PPD.

**Figure A1:**
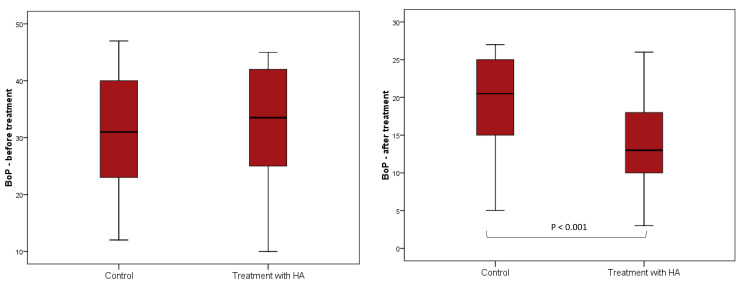
BoP values before (**left**) and after (**right**) treatment in the groups.

**Figure A2:**
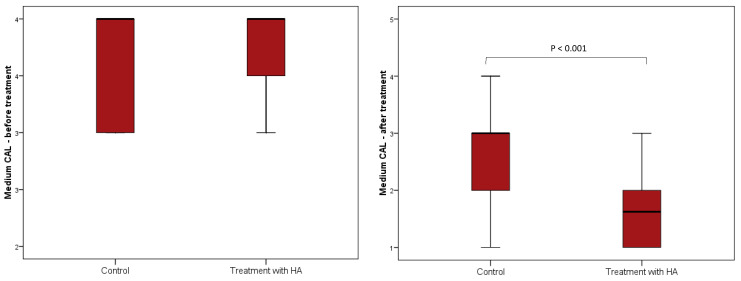
CAL values before (**left**) and after (**right**) treatment in the groups.

**Figure A3:**
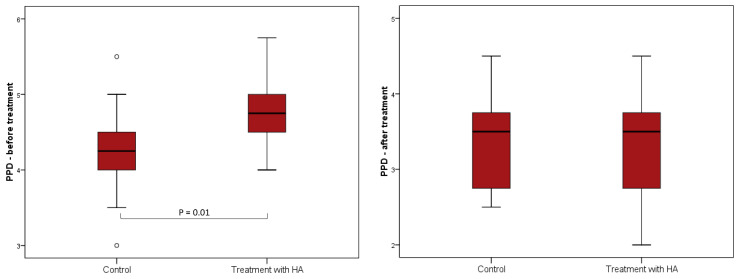
PPD values before (**left**) and after (**right**) treatment in the groups.

In both groups, the observed differences between the parameters tested before and after designated treatment were significant, and revealed the reduction in BoP, CAL, and PPD in both treatment groups (Table 2, Figure A1, Figure A2 and Figure A3). However, when the absolute differences between the two treatment protocols regarding reduction in BoP, CAL, and PPD were tested, it was found that all three parameters were significantly more reduced in the HA group (Table 3).

The effects of specific predictors on the variability in BoP, CAL, and PPD were analyzed by a multivariate regression analysis (stepwise method). No significant predictors were found for CAL or PPD values after treatment for either group. However, in the group of patients receiving therapy without the addition of HA, fewer male participants demonstrated bleeding on probing after treatment compared to their female counterparts (19% vs. 23%, respectively). This was statistically significant at *p* = 0.04, R^2^adj = 0.588. Regarding the patients’ ages, no significant differences were observed, except for the tendency of older participants toward greater PPD values before treatment (Spearman coefficient of correlation (*p* = 0.03).

## 4. Discussion

Non-surgical periodontal therapy is, in the majority of cases, efficacious, and leads to significant improvements in clinical outcomes; however, in some cases, it fails to halt the disease process, which continues to persist. Van Dyke [26] and Salvi and Lang [27] suggest that “resolution of established inflammation takes longer to subside, or may even fail to do so when the inflammation has become chronic, therefore administration of pharmacological or bioactive agents as adjuncts may facilitate resolution or inhibit inflammation”. Commonly used adjuncts in periodontal therapy come in the form of disinfectants (such as chlorhexidine, boric acid, and povidone–iodine), low-level laser therapy, herbal medicine, probiotics, host modulators such as statins, bisphosphonate and metformin gels, antibiotics administered either locally or systemically, and even orthodontic therapy. This list would be incomplete without the most natural of them all: hyaluronic acid.

In the field of dentistry, hyaluronic acid was first used in preliminary clinical trials by Pagnacco and Vangelisti in 1997 [28]. In subsequent trials, this biological macromolecule was found to have clinically proven anti-inflammatory, anti-edematous, anti-bacterial, and pro-angiogenetic properties [29], while some authors also discussed its significant antioxidant capacity, achieved through scavenging of reactive oxidative species, called ROS [30,31]. There are numerous, relevant, published studies and a few meta-analyses that demonstrated positive results in patients with gingivitis, chronic periodontitis, implant, and sinus-lift procedures, as well as oral ulcer treatment [31]. Unfortunately, there are very few hyaluronic-acid-based products that have been registered and tested in randomized clinical trials for applications in non-surgical periodontal therapy and surgical therapy; furthermore, there are some investigations that failed to report the exact product used, making it impossible to determine the type and molecular weight of hyaluronic acid. In this trial, a well-researched product with defined molecular properties and established on the global market was used, with previously proven pre-clinical [32,33] and clinical effects [34,35]. This product is also widely available and proven to be safe for both non-surgical and surgical applications in dentistry.

In this single-center, randomized, controlled study, a total of 100 periodontitis patients divided into two groups (experimental and control) were treated with non-surgical periodontal therapy, according to the EFP guidelines, and followed-up for 3 months. According to the recommendations for evaluation of the results obtained by non-surgical periodontal therapy, this follow-up period is deemed as adequate [29]. One of the groups received an adjunct to scaling and root planning (SRP) in the form of hyaluronic acid gel, and the results were evaluated clinically. Clinical parameters (BoP, PPD, and CAL) showed statistically significant improvements at three months after treatment in both groups, proving the efficacy of the SRP concept. However, the experimental group, which also received HA gel applied directly into the periodontal pockets, showed a significantly lower percentage of sites with bleeding on probing (BoP), a marker of inflammation, as well as lower clinical attachment loss (CAL), which is indicative of periodontal reparation and regeneration; furthermore, the group receiving the HA gel showed significantly greater reduction in all three parameters when absolute differences were tested before and after therapy. As for the values of probing depth (PPD), even though PPD values were significantly more reduced in the HA group, no statistically significant differences were demonstrated for the median PPD value after therapy. This may be explained by the difference in median PPD values at baseline, where the experimental group (the one receiving the adjunctive HA therapy) had significantly higher PPD values. Both study groups at follow-up had a median value of 3.5 mm, which, according to the most recent EFP and American Academy of Periodontology guidelines, correlates with Stage I of periodontitis, which marks the minimum severity of the disease. However, it should be noted that the CAL value is preferred in clinical trials as the primary outcome measure over PPD, since it represents a more objective measure of periodontitis progression and activity [36].

Hereby, the presented results are in line with most similar trials, which demonstrated a favorable effect of HA as an adjunct to non-surgical periodontal treatment. Many of these trials aimed at treatment of gingivitis, which does not present with the loss of clinical attachment [37,38]. One recently published meta-analysis found a total of 11 RCTs evaluating the effect of this biological macromolecule on healing after its use in treatment [29]; however, only five of them met the selected inclusion criteria and included the measures of BoP, CAL, and PPD. The calculated weighed mean differences for BoP, PPD, and CAL before and after therapy were all in favor of the HA treatment protocol, which is in line with the results obtained in this investigation. Interestingly, one of the RCTs included in the above-mentioned meta-analysis found no differences in terms of BoP, CAL, or PPD values between the control and experimental groups; however, the sulcus fluid flow rate had reduced to physiological levels faster in the HA group [39]. One of the trials did not use any of the commercially available HA gels used nowadays, but a mix of amino acids and sodium hyaluronate gel of unknown molecular weight and concentration [40]. Of the remaining three RCTs, Eick et al. showed greater PPD reduction compared to our results [41], as well as Johannsen et al., who found almost no CAL gain after 12 weeks [42]; finally, Wan, in his thesis, only found significant differences in terms of BoP, and not for PPD or CAL [43].

It can be deduced from the available scientific evidence that the addition of hyaluronic acid to standard, non-surgical, periodontal therapy definitely has some positive biological effects. One of the recently published in vitro studies demonstrated that HA increased the expression of genes encoding type III collagen and transforming growth factor-β3, and subsequently enhanced pro-proliferative, pro-migratory, and pro-inflammatory factors in fibroblasts [44]. In addition to its bacteriostatic effect, it seems obvious that this biological macromolecule is one of the more promising adjuncts to regenerative therapy and is definitely here to stay.

Even though this investigation demonstrated a significant beneficial effect of hyaluronic acid on periodontal healing and reduction in inflammation after the non-surgical periodontal therapy, we were not able to establish whether this remained true in the long run. The patients were followed-up for three months, and it would be very interesting to observe the long-term clinical effects after six- or even twelve-month intervals. Another limitation of this study is the observed difference in median PPD values between the two groups at baseline—the HA group had deeper periodontal pockets. However, by calculating the absolute difference between the groups before and after therapy, we were able to demonstrate that addition of HA significantly improved all parameters used as measures of periodontal disease activity.

It may also be argued that a split-mouth protocol would have greater strength, since it could eliminate the cross-over effects related to parallel groups. However, in our opinion, application of hyaluronic acid on one side of the mouth inevitably leads to even its slight spread among other oral tissues, since it is carried away and spread by saliva; another observation related to the study design is that even split-mouth trials, such as the one by Rajan et al. [45], found the same beneficial effects of hyaluronic acid, such as the ones presented in this paper. Therefore, it seems that both study designs are valid. The future guidelines on adjuncts to periodontal therapy, which may eventually be drafted by relevant institutions and associations, should have the scope of defining the make-up and exact concentrations of hyaluronic acid gels designated for use in the oral cavity, specifically in periodontology. Only then we will be able to grasp the entire array of effects demonstrated by this quite fascinating biological macromolecule.

## 5. Conclusions

Within the limits, the hereby presented data from the randomized controlled trial indicated that the addition of hyaluronic acid to periodontal pockets immediately upon completion of the initial (non-surgical) periodontal therapy leads to significant clinical benefits. Those benefits manifest predominantly through a greater gain in clinical attachment and reduced bleeding on probing, both of which are indicative of reduced inflammation and periodontal regeneration. Hyaluronic acid is an easy-to-handle, safe, biocompatible, non-allergenic, naturally occurring macromolecule with promising clinical effects. However, further long-term studies (of six, twelve, or even more months), possibly with repeated applications of HA at different time intervals, are needed to investigate whether these favorable effects remain over time.

## Figures and Tables

**Table 1:** Clinical parameters before and after therapy in the study and control groups.

	Control Group	Study HA Group	Difference ^†^ (95% CI)	*p* *
**Before therapy**				
BoP (%)	31 (22.8–40.3)	33.5 (23.8–42)	0 (−4 to 5)	0.79
CAL (mm)	4 (3–4)	4 (3.5–4)	0 (0–0)	0.90
PPD (mm)	4.25 (4–4.5)	4.75 (4.4–5)	0.25 (0–0.5)	0.001
**After therapy**				
BoP (%)	20.5 (15–25)	13 (9.5–18.25)	−6 (−10 to −3)	<0.001
CAL (mm)	3 (2–3)	1.63 (1–2)	−1 (−1.25 to −1)	<0.001
PPD (mm)	3.5 (2.8–3.8)	3.5 (2.75–3.75)	0 (−0.25 to 0.25)	0.70

HA—hyaluronic acid; 95% CI—95% confidence interval; * Mann–Whitney U test; ^†^ Hodges–Lehmanov median difference.

**Table 2:** Clinical parameters before and after treatment, within each treatment group.

	Median (Interquartile Range)		Difference ^†^ (95% CI)	*p* *
	Before Treatment	After Treatment		
**Control Group**				
BoP (%)	31 (22.8–40.3)	20.5 (15–25)	−12 (−14 to −9.5)	<0.001
CAL (mm)	4 (3–4)	3 (2–3)	−1 (−1.13 to −1)	<0.001
PPD (mm)	4.25 (4–4.5)	3.5 (2.8–3.8)	−1 (−1.13 to −0.88)	<0.001
**Study HA Group**				
BoP (%)	33.5 (23.8–42)	13 (9.5–18.25)	−18 (−21 to −14.5)	<0.001
CAL (mm)	4 (3.5–4)	1.63 (1–2)	−2.25 (−2.5 to −2)	<0.001
PPD (mm)	4.75 (4.4–5)	3.5 (2.75–3.75)	−1.5 (−1.63 to −1.25)	<0.001

HA—hyaluronic acid; 95% CI—95% confidence interval; * Wilcoxon test; ^†^ Hodges–Lehman median difference.

**Table 3:** Absolute differences before and after therapy between groups.

	Median (Interquartile Range) of Difference Before–After Therapy		Difference ^†^ (95% CI)	*p* *
	Control Group	Study HA Group		
BoP (%)	−11.5 (−18 to −6)	−17 (−26 to −10)	−6 (−10 to −2)	0.003
CAL (mm)	−1 (−1.25 to −0.5)	−2 (−3 to −2)	−1 (−1.5 to −1)	<0.001
PPD (mm)	−1 (−1.25 to −0.75)	−1.5 (−1.75 to −1)	−0.5 (−0.5 to −0.25)	<0.001

95% CI—95% Confidence interval; * Mann–Whitney U test; ^†^ Hodges–Lehmann median difference.

## Data Availability

The data presented in this study are available on request from the corresponding author. The data are not publicly available due to ethical restrictions.
